# Perinatal nicotine exposure induces asthma in second generation offspring

**DOI:** 10.1186/1741-7015-10-129

**Published:** 2012-10-30

**Authors:** Virender K Rehan, Jie Liu, Erum Naeem, Jia Tian, Reiko Sakurai, Kenny Kwong, Omid Akbari, John S Torday

**Affiliations:** 1 Department of Pediatrics, Los Angeles Biomedical Research Institute at Harbor-UCLA Medical Center at David Geffen School of Medicine, 1124 West Carson Street, Torrance, 90502, USA; 2 Department of Molecular Microbiology and Immunology, University of Southern California, Keck School of Medicine, 1975 Zonal Avenue, Los Angeles, 90033, USA

**Keywords:** nicotine, lung, epigenetic, asthma, multigenerational, gender difference

## Abstract

**Background:**

By altering specific developmental signaling pathways that are necessary for fetal lung development, perinatal nicotine exposure affects lung growth and differentiation, resulting in the offsprings' predisposition to childhood asthma; peroxisome proliferator-activated receptor gamma (PPARγ) agonists can inhibit this effect. However, whether the perinatal nicotine-induced asthma risk is restricted to nicotine-exposed offspring only; whether it can be transmitted to the next generation; and whether PPARγ agonists would have any effect on this process are not known.

**Methods:**

Time-mated Sprague Dawley rat dams received either placebo or nicotine (1 mg/kg, s.c.), once daily from day 6 of gestation to postnatal day (PND) 21. Following delivery, at PND21, generation 1 (F1) pups were either subjected to pulmonary function tests, or killed to obtain their lungs, tracheas, and gonads to determine the relevant protein markers (mesenchymal contractile proteins), global DNA methylation, histone 3 and 4 acetylation, and for tracheal tension studies. Some F1 animals were used as breeders to generate F2 pups, but without any exposure to nicotine in the F1 pregnancy. At PND21, F2 pups underwent studies similar to those performed on F1 pups.

**Results:**

Consistent with the asthma phenotype, nicotine affected lung function in both male and female F1 and F2 offspring (maximal 250% increase in total respiratory system resistance, and 84% maximal decrease in dynamic compliance following methacholine challenge; *P *< 0.01, nicotine versus control; *P *< 0.05, males versus females; and *P *> 0.05, F1 versus F2), but only affected tracheal constriction in males (51% maximal increase in tracheal constriction following acetylcholine challenge, *P *< 0.01, nicotine versus control; *P *< 0.0001, males versus females; *P *> 0.05, F1 versus F2); nicotine also increased the contractile protein content of whole lung (180% increase in fibronectin protein levels, *P *< 0.01, nicotine versus control, and *P *< 0.05, males versus females) and isolated lung fibroblasts (for example, 45% increase in fibronectin protein levels, *P *< 0.05, nicotine versus control), along with decreased PPARγ expression (30% decrease, *P *< 0.05, nicotine versus control), but only affected contractile proteins in the male trachea (*P *< 0.05, nicotine versus control, and *P *< 0.0001, males versus females). All of the nicotine-induced changes in the lung and gonad DNA methylation and histone 3 and 4 acetylation were normalized by the PPARγ agonist rosiglitazone except for the histone 4 acetylation in the lung.

**Conclusions:**

Germline epigenetic marks imposed by exposure to nicotine during pregnancy can become permanently programmed and transferred through the germline to subsequent generations, a ground-breaking finding that shifts the current asthma paradigm, opening up many new avenues to explore.

## Background

Asthma is a major public health problem [1,2]. It is the most common chronic disease of childhood [3,4], resulting in a significant medical burden and the resultant healthcare costs [5,6]. The burden of this disease is increasing rapidly, with the most striking increases seen among children, the prevalence rates in some populations being greater than 30% [7]. Although a multitude of causes contribute to childhood asthma, maternal smoking during pregnancy is a well-established contributor [8-13], and it is a major modifiable risk factor, the elimination of which could significantly reduce the prevalence of childhood asthma. However, given that the tobacco industry continues to spend billions of dollars on advertising to attract young smokers, it is unlikely that the problem of smoking during pregnancy will go away any time soon [14]. Approximately 250,000,000 women smoke daily world-wide. Twelve percent of US women still continue to smoke during pregnancy, resulting in the births of at least 400,000 smoke-exposed infants per year in the US [15,16]. This aspect of the smoking-induced asthma etiology is particularly important since there is emerging evidence that, following *in utero *exposure to maternal smoke, asthma can be transmitted multigenerationally. For example, a questionnaire in the Children's Health Study from Southern California reported that grandmaternal smoking during pregnancy increases the risk of asthma in grandchildren regardless of the presence of maternal smoking [17]. Yet there is neither experimental evidence nor any mechanistic explanation for this phenomenon. Using a well-established rat model of *in utero *nicotine exposure for childhood asthma [18-20], we aimed to determine if *in utero *nicotine exposure would transmit asthma to the second generation offspring, and if epigenetic mechanism(s) could be involved in this transmission.

The mechanism linking smoke/nicotine exposure during pregnancy to multigenerational (MG) transmission of asthma is unknown. Since transmission via the germline is the most likely explanation for the non-genetic MG transmission phenomenon, we rationalized that epigenetic modifications of the germline explain MG transmission of perinatal nicotine-induced asthma. For example, a change in the organism's environment, that is, smoke/nicotine exposure (of the F0 generation), can result in modifications in gene expression through alterations in DNA methylation, histone (H) modification, non-coding RNA, and/or protein structure and assembly without changing the DNA sequence of the F1 germline, which can be transmitted to subsequent generations [21,22]. However, whether epigenetic marks such as alterations in DNA methylation and/or H modifications acquired in one generation can be inherited by the next generation is not clear. While one study reported that the acquired DNA methylation marks are not transmitted to subsequent generations [23], another study came to the opposite conclusion [24]. We hypothesize that smoke/nicotine-induced epigenetic marks can become permanently programmed and transferred through the germline to subsequent generations, resulting in altered phenotypic changes at the cellular and consequent organismal levels (for example, asthma in the case of *in utero *smoke/nicotine exposure) in the offspring over multiple subsequent generations, for example, F2, F3, and so on.

Furthermore, the effect of the child's gender on asthma risk following perinatal exposure to cigarette smoke is not clear since an increased risk in both boys [25] and girls [26] has previously been reported. However, since there is emerging evidence to suggest that finely-tuned developmental programs, such as that of the lung, may be sensitive to specific environmental challenges in a sex-specific manner, particularly during the developmental programming and gametogenesis stages [27], and since maternal smoking is known to affect fetal growth more profoundly in the male fetus [28], we also hypothesized that there would be a sexual dimorphism in asthma risk following *in utero *smoke exposure, with males being more susceptible than females.

## Methods

### Materials

Nicotine bitartrate was acquired from Sigma-Aldrich (St. Louis, MO, USA) and rosiglitazone (RGZ) from Cayman Chemical (Chicago, IL, USA). DNA methylation (cat#: P-1034-96), H3 (cat#: P-4008-96) and H4 (cat#: P-4009-96) acetylation kits were obtained from Epigentek (Farmingdale, NY, USA). All plasticware and culture media were purchased from Corning (Corning, NY, USA) and Invitrogen, Inc. (San Deigo, CA, USA).

### Animal model (Schematic 1)

Pathogen-free timed (embryonic day 0 = day of mating) pregnant Sprague-Dawley F0 rats (200 to 250 g body weight) were obtained from Charles River (Hollister, CA, USA), and allowed to acclimatize until embryonic day 6 [see Additional File 1 for Schematic]. Dams were randomized to receive placebo (diluent, normal saline), nicotine (1 mg/kg, s.c.) alone, or nicotine (1 mg/kg), and the PPARγ agonist RGZ (3 mg/kg, i.p.) in 100 μL volumes once daily from embryonic day 6 to postnatal day (PND) 21. The dams were allowed free access to water and pair-fed according to the previous day's intake by the nicotine group animals and were maintained in a 12:12-hour light-dark cycle. Following delivery at term, the F1 pups were allowed to breast feed *ad libitum*. At PND21 the pups were either subjected to pulmonary function tests (PFTs) and then killed, or killed and their lungs and tracheas collected for airway contractility protein levels, fibroblast isolation, and tracheal tension studies. The gonads were collected to determine epigenetic marks. Some F1 animals were weaned at PND21 and maintained as breeders to generate F2 rats, but without any exposure to nicotine in the F1 pregnancy. At PND21, F2 animals underwent studies similar to those performed on F1 animals. All studies were approved by the Los Angeles Biomedical Research Institutional Review Board and were conducted in accordance with the National Institutes of Health Guide for the Care and Use of Laboratory Animals.

The dose (1 mg/kg) chosen for the nicotine treatment has previously been shown to result in an asthma phenotype in the offspring in a number of studies (reviewed in [18]) and is comparable to the dose of nicotine to which habitual smokers are exposed, that is, approximately 1 mg/kg/body weight [29]. As further support for the doses of nicotine and RGZ used in this study, pulmonary function changes induced following perinatal exposure to 1 mg/kg/day nicotine, administered s.c., were recently shown to be blocked by the concomitant i.p. administration of 3 mg/kg/day RGZ (20). It is also important to note that this dose of RGZ has previously been shown to block hyperoxia-induced neonatal lung injury as well [30]. Two animals were used for each condition per experiment, and each experiment was repeated at least three times.

### Lung fibroblast isolation

PND21 rat lung fibroblasts were cultured according to previously described methods, with slight modifications [30]. Briefly, the lungs were removed and put into Hanks' balanced salt solution (HBSS) and chopped into small pieces. The HBSS was decanted and 5 volumes of 0.05% trypsin were added to the lung preparation. The lungs were dissociated in a 37°C water bath using a Teflon stirring bar to disrupt the tissue mechanically. Once the tissue was dispersed into a unicellular suspension, the cells were pelleted at 500 × *g *for 10 minutes at room temperature in a 50-mL polystyrene centrifuge tube. The supernatant was decanted, and the pellet was resuspended in minimal essential medium (MEM) containing 10% FBS to yield a mixed cell suspension of approximately 3 × 10^8 ^cells, as determined using a Coulter particle counter (Beckman-Coulter, Hayaleah, FL, USA). The cell suspension was then added to culture flasks (75 cm^2^) for 30 to 60 minutes to allow for the differential adherence of lung fibroblasts. These cells are greater than 95% pure fibroblasts based upon vimentin-positive staining.

### Pulmonary function testing

Measurement of respiratory function was performed with a plethysmograph for restrained animals (Buxco Inc, Troy, NY, USA); the pups were deeply anesthetized and sedated with ketamine (70 mg/kg, Bioniche Teoranta Inverin, Co., Galway, Ireland), and xylazine (7 mg/kg, Akorn, Inc., Decatur, IL, USA), tracheostomized and ventilated. Rats were exposed to increasing concentrations of aerosolized methacholine (0, 1.25, 2.5, 5, 10, and 20 mg/ml) over a period of 3 minutes. Lung resistance (Rrs) and dynamic compliance (Cdyn) were plotted as a function of the methacholine concentration administered.

### Tracheal constriction studies

The trachea was excised *en bloc *immediately after sacrifice and dissected free of connective tissue in ice-cold modified Krebs-Ringer bicarbonate buffer (in mM: 118.3 NaCl, 4.7 KCl, 2.5 CaCl_2_, 1.2 MgSO_4_, 1.2 KH_2_PO_4_, 25.0 NaHCO_3 _and 11.1 glucose). Subsequently, an approximately 6 mm tracheal ring was resected from the midsection and used for tracheal tension studies. The tracheal ring was suspended in an organ chamber filled with 10 ml of modified Krebs-Ringer bicarbonate solution maintained at 37 ± 0.5°C and aerated with 95% O_2_-5% CO_2 _(pH 7.4). Each ring was suspended via two stirrups that were passed through the lumen: one stirrup was anchored to the bottom of the organ chamber and the other stirrup was connected to a strain gauge (model FT03C, Grass Instrument, Quincy, MA, USA) for the measurement of isometric force, as described previously [20].

At the beginning of the experiment, each tracheal ring was stretched to its optimal resting tension, which was achieved by step-wise stretching in 0.1-g increments until the contractile response to 100 mM KCl reached a plateau. The optimal resting tension was measured, and then each tracheal ring was allowed to equilibrate for one hour after it was brought to its optimal resting tension. The effects of acetylcholine were determined at least 30 minutes after the administration of nitro-L-arginine (1 × 10^-4 ^M, an inhibitor of nitric oxide synthase). In all experiments, indomethacin (1 × 10^-5 ^M) was added to the bath to prevent the possible interference by prostanoids.

### Western blot

Western blot analysis for fibronectin, α-smooth muscle actin (α-SMA), calponin, collagens I and III, nicotinic acetylcholine receptors α3 and α7, and PPARγ were performed as described previously [20].

### Real Time Reverse Transcription-Polymerase Chain Reaction

RT-PCR was performed as previously described [19,31].

### Global DNA methylation

Genomic DNA from lungs, testes, and ovaries was isolated using QIAamp DNA Mini Kit (Qiagen, Valencia, CA, USA, Cat. No.: 51304) and the DNA concentration was determined using a NanoDrop2000. Global DNA methylation was quantified using a MethylFlash Methylated DNA Quantification Kit (Epigentek, Cat. No.: P-1034) according to the manufacturer's instructions. Briefly, DNA is bound to strip wells that are specifically treated to have high DNA affinity. The methylated fraction of DNA is detected using capture and detection antibodies, and then quantified colorimetrically by reading the absorbance at 450 nm in a Wallac 1420 Multilabel Counter. The amount and percentage of methylated DNA (5-mC) in the total DNA extract was calculated based on a standard curve generated using a methylated DNA positive control.

### Global histone H3 and H4 acetylation

Histone protein from lungs, testes, and ovaries was extracted using an EpiQuik Total Histone Extraction Kit (Epigentek, Cat. No.: OP-0006), and histone protein concentration was measured by the Bradford method, using BSA as the standard. Global histone H3 and H4 acetylation was quantified using an EpiQuik Global Histone H3 Acetylation Assay Kit (Epigentek, Cat. No.: P-4008) and an EpiQuik Global Histone H4 Acetylation Assay Kit (Epigentek, Cat. No.: P-4009) according to the manufacturer's instructions. Briefly, the histone proteins are stably spotted on the strip wells. The acetylated histone H3 and H4 are recognized with high-affinity antibodies against them. The amount of acetylated H3 and H4 are quantified through a horseradish peroxidase-conjugated secondary antibody-color development system, and the absorbance at 450 nm is read in a Wallac 1420 Multilabel Counter. The amount of acetylated histone H3 and H4 in the total histone protein was calculated according to a standard curve generated using acetylated histone H3 or H4.

### Statistics

The data for analysis were obtained from at least three independent sets of experiments. Analysis of variance (ANOVA) for multiple comparisons with Bonferroni *post-hoc *analysis and Student's t-test, as indicated, were used, and *P *< 0.05 was considered to indicate significant differences among the experimental groups.

## Results

**Figure 1:**
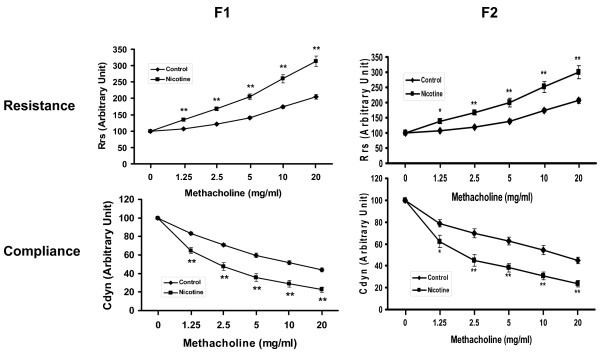
**Effect of perinatal nicotine exposure on mixed gender offspring pulmonary function**. Compared to the control group, with nicotine administration there was a significant increase in total airway resistance and a decrease in total compliance following Mch challenge in both F1 and F2 rats of mixed gender even though the F2 rats were not exposed to any nicotine during the F1 gestation. Values are means ± SE. n = 10 to 12 for each group. *P < 0.05, **P < 0.01, versus control. Mch, methacholine.

**Figure 2:**
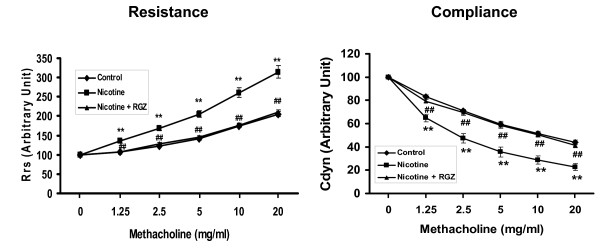
**Effect of rosiglitazone (RGZ) on perinatal nicotine exposure-induced alterations in mixed gender offspring pulmonary function**. Compared to the control group, with nicotine administration there was a significant increase in resistance and a significant decrease in compliance of the lung following a Mch challenge, both of which were blocked by concomitant RGZ administration. Values are means ± SE. n = 10 to 12 for each group. **P < 0.01, versus control; ##*P *< 0.01, versus nicotine group. Mch, methacholine.

We initially determined the effect of nicotine on pulmonary function in response to a graded methacholine challenge. Compared to the control group, with perinatal nicotine exposure to F0 dams (Figure 1) there was a significant increase in total airway Rrs, and a decrease in total airway Cdyn of the respiratory system, not only in F1 rats [20], but also in F2 rats (*P *< 0.01 versus control for both Rrs and Cdyn), even though the F2 rats were not exposed to nicotine. In accord with our recently published data on F1 rats [20], RGZ treatment blocked the nicotine-induced increase in Rrs and decrease in Cdyn in F2 rats (*P *< 0.01 versus nicotine for both Rrs and Cdyn) (Figure 2).

**Figure 3:**
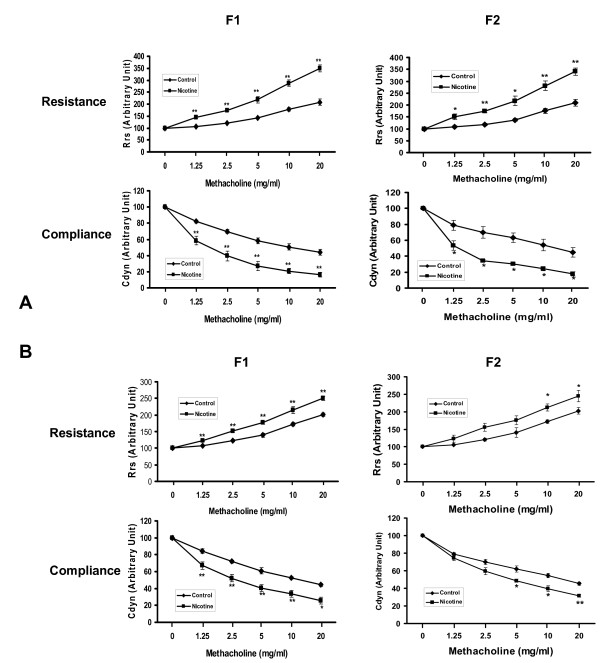
**Effect of perinatal nicotine exposure on male and female offspring pulmonary function**. Compared to the control group, with nicotine administration there were significant increases in resistance and decreases in compliance of the lung following Mch challenge in F1 and F2 male (**A**) and female (**B**) rats even though the F2 pups were not exposed to any nicotine during gestation. Values are means ± SE. n = 5 to 6 for each group. *P < 0.05, **P < 0.01 versus control. Mch, methacholine.

We subsequently analyzed the effects of nicotine exposure on PFT's in male and female offspring separately (Figure 3a and 3b). Following a methacholine challenge, perinatal nicotine exposure only to F0 dams caused a significant increase in Rrs and a decrease in Cdyn in both the males and females of the F1 and F2 generations (*P *< 0.05, males versus females, for both Rrs and Cdyn).

**Figure 4:**
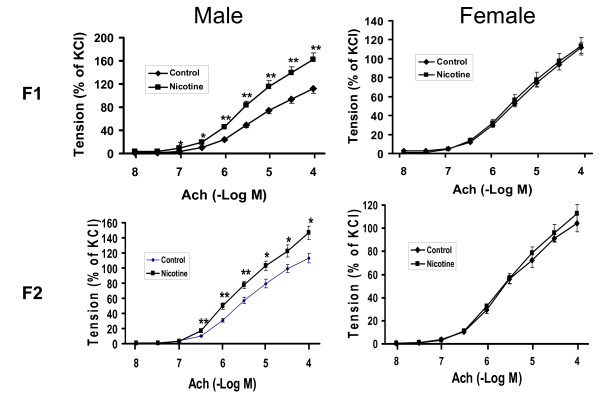
**Effect of perinatal nicotine exposure on acetylcholine tracheal constriction response in F1 and F2 offspring**. Compared to the control group, with nicotine administration there was a significant increase in tracheal constriction in response to acetylcholine only in the males of both the F1 and F2 generations. Values are means ± SE; * *P *< 0.05, ** *P *< 0.01, versus control group; n = 5 to 6.

Having determined the effect of *in utero *nicotine exposure on the F1 and F2 offspring whole respiratory systems, we examined the effect of nicotine on tracheal rings isolated from F1 and F2 offspring (Figure 4). Nicotine administration caused a significant increase in tracheal constriction in response to acetylcholine only in the males of both the F1 and F2 generations (*P *< 0.01 versus control).

**Figure 5:**
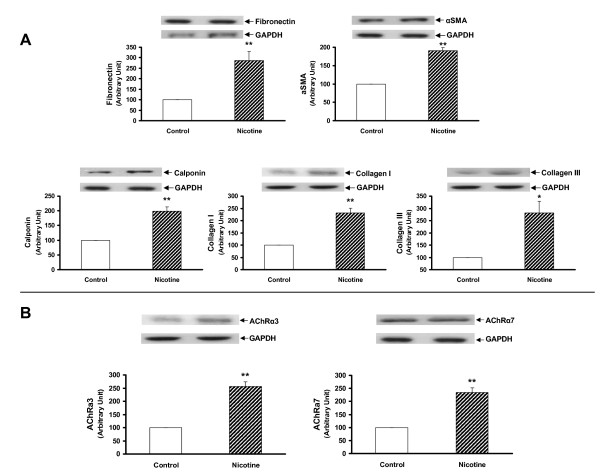
**Effect of perinatal nicotine exposure on mesenchymal protein markers of airway reactivity and nicotinic acetylcholine receptors α3 and α7 in lungs of male F2 rats**. Compared to the control group, with nicotine administration the protein levels of fibronectin, α-SMA, calponin, and collagens I and III (**A**) and nicotinic acetylcholine receptors α3 and α7 (**B**) increased significantly. Upper panels show representative Western blots for these markers and glyceraldehyde 3-phosphate dehydrogenase (GAPDH). Lower panels show the densitometric values of the markers normalized to GAPDH. Values are means ± SE. n = 6 for each group. * *P *< 0.05, **P < 0.01, versus control group. α-SMA, α-smooth muscle actin.

**Figure 6:**
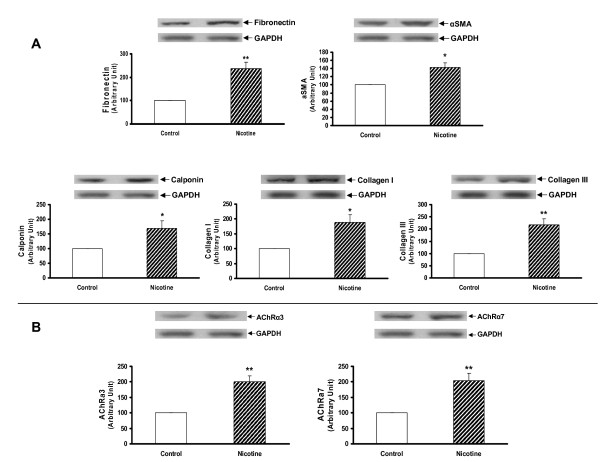
**Effect of perinatal nicotine exposure on mesenchymal protein markers of airway reactivity and nicotinic acetylcholine receptors α3 and α7 in lungs of female F2 rats**. Compared to the control group, with nicotine administration the protein levels of fibronectin, α-SMA, calponin, and collagens I and III (**A**) and nicotinic acetylcholine receptors α3 and α7 (**B**) increased significantly. Upper panels show representative Western blots for the markers and GAPDH. Lower panels show the densitometric values for these markers normalized to GAPDH. Values are means ± SE. n = 6 for each group. * *P *< 0.05, **P < 0.01, versus control group. GAPDH, glyceraldehyde 3-phosphate dehydrogenase; α-SMA, α-smooth muscle actin.

**Figure 7:**
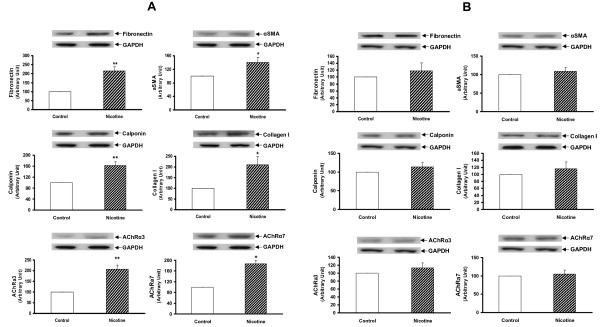
**Effect of perinatal nicotine exposure on mesenchymal protein markers of airway reactivity and nicotinic acetylcholine receptors α3 and α7 in tracheas of male and female F2 rats**. Compared to the control group, with nicotine administration the protein levels of fibronectin, α-SMA, calponin, and collagens I and III increased significantly in male (**A**) but not in female rats (**B**). Upper panels show representative Western blots for these markers and GAPDH. Lower panels show the densitometric values of these markers normalized to GAPDH. Values are means ± SE. n = 6 for each group. * *P *< 0.05, **P < 0.01, versus control group. GAPDH, glyceraldehyde 3-phosphate dehydrogenase; α-SMA, α-smooth muscle actin.

**Figure 8:**
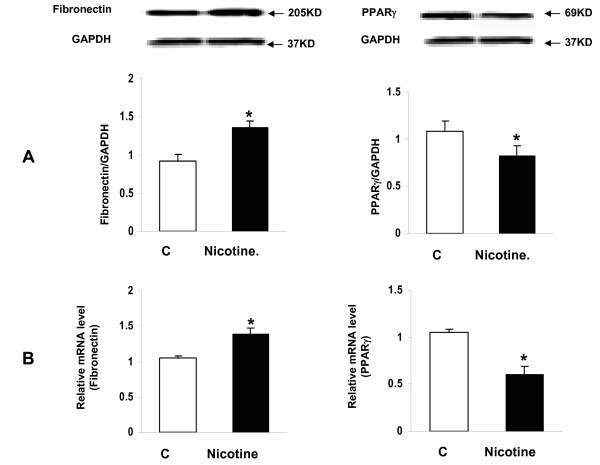
**Effect of perinatal nicotine exposure on markers of differentiation in fibroblasts isolated from the lungs of F2 rats (mixed gender)**. Compared to the control group, with nicotine administration the expression of fibronectin increased and that of PPARγ decreased, at both the protein (**A**) and mRNA (**B**) levels. Values are means ± SE. n = 3 for each group. * *P *< 0.05, versus control group. GAPDH, glyceraldehyde 3-phosphate dehydrogenase; PPARγ, peroxisome proliferator-activated receptor gamma.

Since we had previously determined that the nicotine exposure of F0 dams increased the expression of lung contractile proteins and inhibited PPARγ expression in both the whole lung and isolated alveolar interstitial fibroblasts of F1 rats [20], we next determined this effect in the F2 offspring. We found that the protein levels of fibronectin, α-SMA, calponin, and collagens I and III, and nicotinic acetylcholine receptors α3 and α7 were all increased significantly in the whole lung lysates of both the male (Figure 5a and 5b) and female F2 (*P *< 0.05 versus control for all) (Figure 6a and 6b) rats. In contrast, at the tracheal level the expression of fibronectin, α-SMA, calponin, collagen I, nicotinic acetylcholine receptors (nAChR) α3 and α7 were increased only in males (*P *< 0.05 versus control) (Figure 7a and 7b). In line with our recently published data, the expression of fibronectin was increased, and that of PPARγ was decreased, at both the mRNA and protein levels in the alveolar interstitial fibroblasts isolated from the lungs of F2 rats (*P *< 0.05 versus control) (Figure 8).

**Figure 9:**
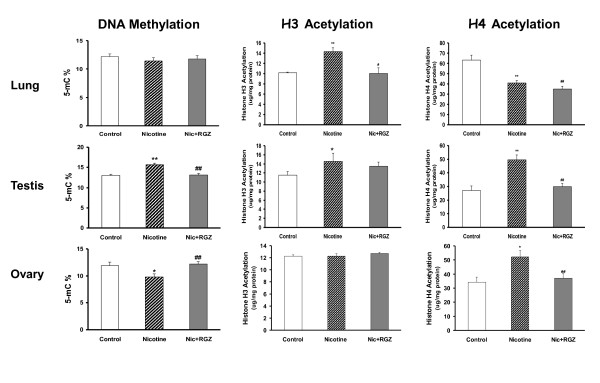
**Effect of rosiglitazone (RGZ) in perinatal nicotine exposure-induced changes in global DNA methylation and histone (H) 3 and 4 acetylation in the lungs and gonads of F1 rats**. Compared to the control group, with nicotine administration the level of global DNA methylation increased in the testes, decreased in the ovaries, but did not change in the lungs (**A**); H3 acetylation increased in the lungs and testes, but did not change in the ovaries (**B**); H4 acetylation decreased in the lungs, while it increased in the testes and ovaries (**C**). Most of these changes were blocked by concomitant RGZ administration. For example, in the lungs the nicotine-induced increase in lung H3 acetylation was blocked by RGZ, whereas there was no effect on global DNA methylation or on the nicotine-induced decrease in H4 acetylation; in the testes, the nicotine-induced increase in DNA methylation and H4 acetylation were blocked, without any effect on H3 acetylation; and in the ovaries, the nicotine-induced decrease in DNA methylation and increase in H4 acetylation were blocked without any effect on H3 acetylation. Values are means ± SE with variable numbers for each group. *P < 0.05, **P < 0.01, versus control; n = 4 to 6.

**Figure 10:**
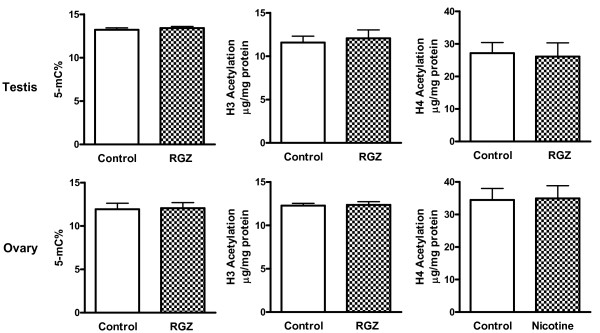
**Effect of perinatal rosiglitazone exposure on global lung and gonadal DNA methylation and histone (H) 3 and 4 acetylation in F1 rats**. With perinatal rosiglitazone exposure alone during gestation there was no effect on global DNA methylation and H3 and H4 acetylation in the lungs and gonads of F1 rat pups. Values are means ± SE; n = 4 to 6.

To determine the likely mechanism of nicotine-induced transmission of asthma to the second generation offspring, we next determined the effect of F0 nicotine exposure on global DNA methylation and histone acetylation in the lungs and gonads of the F1 rats. We observed that with nicotine administration global DNA methylation increased in the testes (*P *< 0.01), decreased in the ovaries (*p *< 0.05), but did not change in the lungs (Figure 9a); H3 acetylation increased in the lungs (*P *< 0.01) and testes (*P *< 0.05), but did not change in the ovaries (Figure 9b); H4 acetylation decreased in the lungs (*P *< 0.01) while it increased in the testes (*P *< 0.01) and ovaries (*P *< 0.05) (Figure 9c). Further, we determined if the nicotine effects on histone methylation and acetylation were affected by RGZ, which has been shown to normalize the structural and functional effects of nicotine on the offspring lung phenotype at F1 [20]. The nicotine-induced increase in lung H3 acetylation was blocked (Figure 9b) by RGZ treatment, whereas there was no effect on global DNA methylation (Figure 9a) or on the nicotine-induced decrease in H4 acetylation (Figure 9c), suggesting a central role of H3 acetylation in mediating nicotine's effects on the pulmonary phenotype. Finally, it is reassuring to note that perinatal exposure of the gonads to RGZ by itself did not result in any significant changes in global gonadal DNA methylation or in H3 and H4 acetylation (Figure 10).

## Discussion

In the present series of experiments we have observed significant effects of nicotine treatment on lung function in the next two generations, affecting both the male and female offspring. In sharp contrast, nicotine treatment only affected the tracheal contractility of the male offspring. The functional effects of nicotine on the naive offspring were accompanied by increased expression of contractile proteins in the whole lung, as well as in the associated isolated lung fibroblasts, accompanied by decreased PPARγ expression. These nicotine-induced changes in lung function and mesenchymal protein expression, accompanied by decreased PPARγ expression, are consistent with the effect of nicotine on myofibroblast differentiation [32]. Even more importantly, along with the normalization of the asthma phenotype in the F1 and F2 offspring, most of the nicotine-induced lung and gonadal epigenetic changes were also normalized. For example, nicotine-induced increases in H3 acetylation in the lung, DNA methylation and H4 acetylation in the testis, and the decrease in DNA methylation and increase in H4 acetylation in the ovaries of F1 offspring were normalized by RGZ treatment, but it had no effect on lung H4 acetylation, providing further mechanistic specificity regarding the nature of the epigenetic mechanism. Given these insights, we will extend these studies to F3 and F4 generation offspring in future studies.

Since ruling out genetic and environmental confounders is extremely difficult in humans, there is only scant evidence for MG epigenetic effects for any condition in humans [33]; and in fact, there is none for asthma. Recently emerging evidence suggests that the phenotype of an individual is the cumulative result of complex interactions between the genotype and its current, past and ancestral environments [27,34]. Therefore, it is logical to speculate a role for ancestral cigarette smoke exposure in the child's asthma predisposition. Yet, as pointed out above, there is no evidence for this, other than the data from the Children's Health Study from Southern California [17].

In contrast to other species, the evidence for fetal programming as a mode of MG transmission of traits in humans is very limited. For example, mothers from the Dutch Hunger Winter who were exposed to famine as fetuses delivered offspring of lower birth weights than those with no fetal exposure to famine, although this was not confirmed in a subsequent study [35]. There is also evidence of increased morbidity and mortality associated with parental and grandparental nutritional status, suggesting a role for fetal programming, possibly via epigenetic mechanisms to account for the MG effects [21,36,37]. In contrast to the very limited data in humans, in a variety of animal models gestational exposure to carcinogens, endocrine disruptors, and other toxins has been shown to have MG effects [38-40].

The present study is groundbreaking in our understanding of the mechanisms potentially involved in the transmission of epigenetic human diseases, which to date have only been speculated, albeit based on strong epidemiologic grounds [41]. The observation that nicotine exposure *in utero *affects the F1 offspring DNA methylation of the testes and ovaries, and that H3 acetylation was increased in the lungs and testes of F1 male offspring in association with male-specific increased tracheal constriction is the first demonstration of an epigenetic effect of nicotine on both gametocytes and somatocytes. Furthermore, the specific inhibition of this effect of nicotine on H3 acetylation by RGZ, which we have previously shown to block the nicotine-induced asthma phenotype in F1 offspring [20], provides a unique molecular genetic insight to the MG mechanism of nicotine action. It must be borne in mind, however, that these offspring's developing gonads had been exposed to nicotine *in utero*, leaving open the possibility that the effect was not a bona fide transgenerational effect, but was rather only MG. However, the transmission of the asthma phenotype to the F2 generation, both structurally and functionally, and its prevention by a specifically-targeted molecular intervention is the first unequivocal demonstration of MG transmission of an epigenetically-mediated effect on the offspring phenotype. The fact that the phenotypic effect was on asthma, a well-recognized epidemiologic example of epigenetic transmission of the cause of a public health epidemic, makes this series of experiments all the more significant and noteworthy. This, and the recent finding that even 'thirdhand smoke' can induce the asthma phenotype [42], portends new and rational ways of thinking about effectively coping with the health hazards that abound all around us [43,44].

At first glance, it might seem surprising that the nicotine effect on the asthma phenotype is sex-specific. However, there is a documented association between gender and airway size, first referred to as dysanapsis by Mead [45], in which he showed that boys and women had self-similar airway structure that was distinctly different from that of men. Those data suggest that androgens may differentially affect airway development. We have previously shown that androgens affect the rate of lung development in rabbits and rats by inhibiting the glucocorticoid-induced differentiation of lung fibroblasts [46], consistent with their effect on lipofibroblast differentiation [47], which determines lung development [48]. The airway narrowing of dysanapsis has also been shown to be associated with asthma [49]. Therefore, androgens may precipitate asthma through a common genetic mechanism, since they, like nicotine, stimulate the Wnt pathway [50] and down-regulate PPARγ expression in lung fibroblasts [18-20,31,32].

The compelling epigenetic data presented here potentially shift the current paradigm for our understanding of childhood asthma, and for the first time implicate epigenetics as the underlying cause for increased MG asthma following *in utero *exposure to maternal smoking. In fact, the data provided herein not only provide novel mechanistic information underlying the MG asthma risk, but also pave the way for studying the molecular mechanisms underlying MG effects for a host of other environmental toxins, agonists and antagonists as well.

It is important to point out that although there are many agents in cigarette smoke that may be detrimental to the developing lung, there is plenty of evidence to support nicotine as the main agent that alters fetal lung development: nicotine crosses the human placenta with minimal biotransformation [51]; it accumulates in fetal blood, maternal milk, amniotic fluid, and several fetal tissues, including the respiratory tract [52-54], and has been shown to have direct effects on pulmonary alveolar epithelial cells and interstitial fibroblasts isolated from the developing lung [32,43,55-60]. Therefore, it is not surprising that nicotine exposure during pregnancy is an extensively utilized and well-accepted model to study the effects of cigarette smoke on the developing lung in general, and on asthma in particular [18,20]. It is also important to emphasize that the main effects of *in utero *nicotine exposure on lung growth and development are due to specific alterations in signaling pathways involved in lung development [18,20,31,32,55-60], rather than being due to irreversible disruption by teratogenic or toxicological effects. This feature offers the opportunity for targeted interventions to modulate the effects of *in utero *nicotine exposure, in contrast to toxic or teratologic effects, which would be unlikely to be effectively blocked by any intervention.

It could be argued that the epigenetic marks in the gonads and lung are epiphenomena, and that the RGZ 'rescue' of the normal lung phenotype is an artifact. However, there is experimental evidence that methylation of H3/H4 results in down-regulation of PPARγ expression [61], mechanistically linking the epigenetic effect of nicotine with decreased lipofibroblast expression [18] and asthma [20].

## Conclusions

From the data included here we conclude that the pulmonary effects of nicotine exposure during pregnancy are not restricted only to the offspring of the exposed pregnancy, but are also transmitted to subsequent generation, possibly through germline epigenetic alterations, and, even more importantly, these effects can be blocked by targeted molecular interventions. Moreover, these data not only provide novel mechanistic information underlying the multigeneration transmission of asthma risk following exposure to maternal smoke during pregnancy, but also set precedence for studying other such environmental toxins that might have multigenerational or transgenerational effects.

## Abbreviations

BSA: bovine serum albumin; Cdyn: dynamic compliance; F0, F1, F2: parent, first, and second generations; FBS: fetal bovine serum; HAT: histone acetyl transferase; HBSS: Hanks' balanced salt solution; HDAC: histone deacetylase; H3, H4: histones 3 and 4; MEM: minimal essential medium; MG: multigenerational; PFT: pulmonary function test; PND: postnatal day; PPARγ: peroxisome proliferator activated receptor gamma; RGZ: rosiglitazone maleate; Rrs: lung resistance; RT-PCR: reverse transcriptase-polymerase chain reaction; α-SMA: α-smooth muscle actin.

## Competing interests

The authors declare that they have no competing interests.

## Authors' contributions

VKR conceived the study, participated in study design, helped and coordinated the acquisition of data and its interpretation, and wrote the first draft the manuscript. JL carried out the animal work, molecular and pulmonary function studies, and made the data figures. EN and JT helped with DNA methylation and histone acetylation studies. RS performed fibroblast isolation and RT-PCR studies. KK helped with pulmonary function studies. OA supervised pulmonary function studies and the interpretation of pulmonary function data. JST helped in data interpretation, study design, and manuscript writing and revising. All authors read and approved the final manuscript.

## Pre-publication history

The pre-publication history for this paper can be accessed here:

http://www.biomedcentral.com/1741-7015/10/129/prepub

## Supplementary Material

Additional File 1 **Rat Model of Multigenerational Nicotine-induced Asthma**. Rats were exposed to nicotine *in utero *(F0) to mimic maternal cigarette smoking. F0 offspring were then mated to generate F1 offspring, which in turn were mated to generate F2 offspring. Lungs, ovaries and testes are shown in black or white to symbolize direct or multigenerational nicotine exposure effects, respectively. Click here for file
