# Supplementary material for: Perinatal nicotine exposure induces asthma in second generation offspring
Source: BMC Med. 2012 Oct 30;10:129. doi: 10.1186/1741-7015-10-129 (PMC3568737; doi:10.1186/1741-7015-10-129)
Supplement: Additional File 1 — Rat Model of Multigenerational Nicotine-induced Asthma. Rats were exposed to nicotine in utero (F0) to mimic maternal cigarette smoking. F0 offspring were then mated to generate F1 offspring, which in turn were mated to generate F2 offspring. Lungs, ovaries and testes are shown in black or white to symbolize direct or multigenerational nicotine exposure effects, respectively. [file 1741-7015-10-129-S1.PDF]

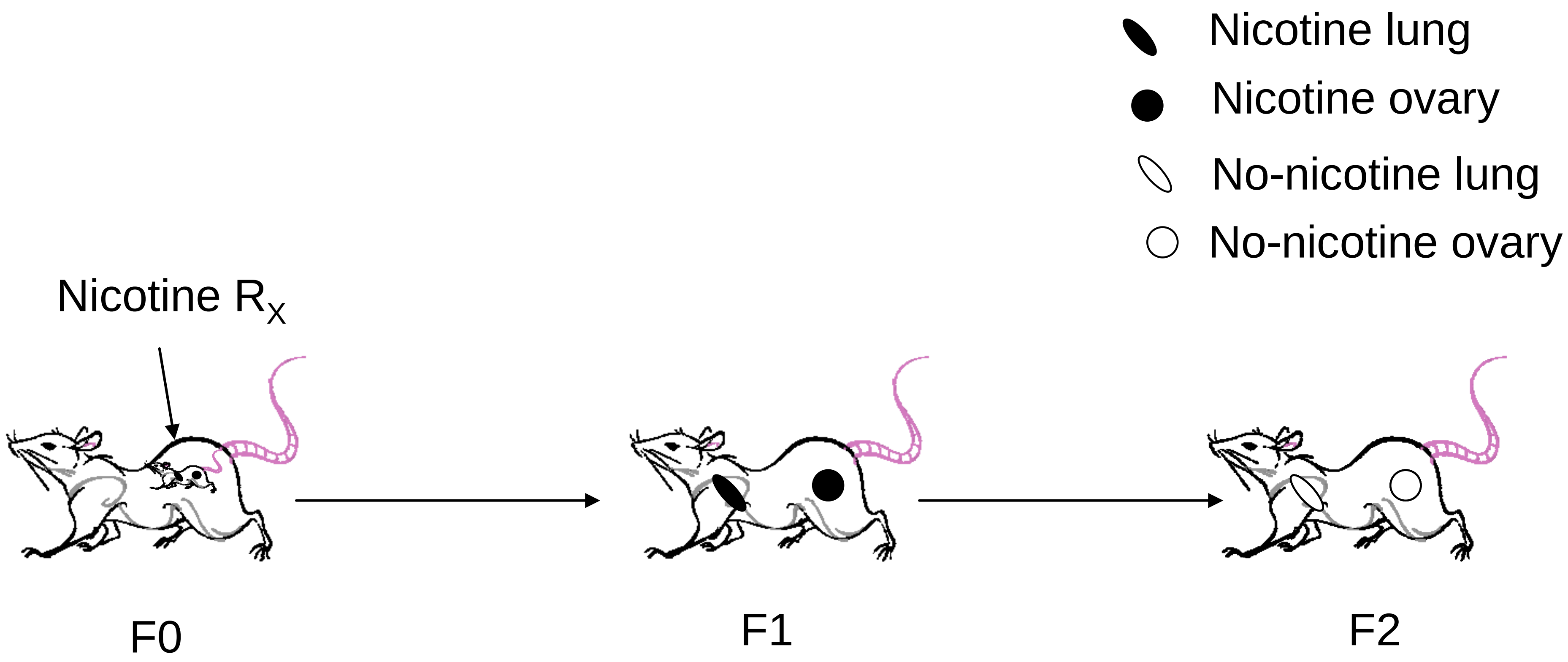

Schematic 1. Rat Model of Multigenerational Nicotine-induced Asthma. Rats were exposed to nicotine in utero (F0) to mimic maternal cigarette smoking. F0 offspring were then mated to generate F1 offspring, which in turn were mated to generate F2 offspring. Lungs, ovaries and testes are shown in black or white to symbolize direct or multigenerational nicotine exposure effects, respectively.
